# Methionine sulfoxide reductase B2 protects against cardiac complications in diabetes mellitus

**DOI:** 10.1186/s13098-024-01390-0

**Published:** 2024-07-05

**Authors:** Seung Hee Lee, Suyeon Cho, Jong Youl Lee, Ji Yeon Kim, Suji Kim, Myoungho Jeong, Jung Yeon Hong, Geun-Young Kim, Seung Woo Lee, Eunmi Kim, Jihwa Kim, Jee Woong Kim, John Hwa, Won-Ho Kim

**Affiliations:** 1 Division of Cardiovascular Disease Research, Department for Chronic Disease Convergence Research, Korea National Institute of Health, Cheongju, Republic of Korea; 2 Division of Endocrine and Kidney Disease Research, Department for Chronic Disease Convergence Research, Korea National Institute of Health, Cheongju, Republic of Korea; 3 Division of Research Support, Department of Research Planning and Coordination, Korea National Institute of Health, Cheongju, Republic of Korea; 4 Yale Cardiovascular Research Center, New Haven, USA

**Keywords:** MsrB2, Diabetes, Cardiac complications, Mitophagy

## Abstract

Diabetes mellitus (DM) is a progressive, chronic metabolic disorder characterized by high oxidative stress, which can lead to cardiac damage. Methionine sulfoxylation (MetO) of proteins by excessive reactive oxygen species (ROS) can impair the basic functionality of essential cellular proteins, contributing to heart failure. Methionine sulfoxide reductase B2 (MsrB2) can reverse oxidation induced MetO in mitochondrial proteins, so we investigated its role in diabetic cardiomyopathy. We observed that DM-induced heart damage in diabetic mice model is characterized by increased ROS, increased protein MetO with mitochondria structural pathology, and cardiac fibrosis. In addition, MsrB2 was significantly increased in mouse DM cardiomyocytes, supporting the induction of a protective process. Further, MsrB2 directly induces Parkin and LC3 activation (mitophagy markers) in cardiomyocytes. In MsrB2, knockout mice displayed abnormal electrophysiological function, as determined by ECG analysis. Histological analysis confirmed increased cardiac fibrosis and disrupted cardiac tissue in MsrB2 knockout DM mice. We then corroborated our findings in human DM heart samples. Our study demonstrates that increased MsrB2 expression in the heart protects against diabetic cardiomyopathy.

**Graphic Abstract:**

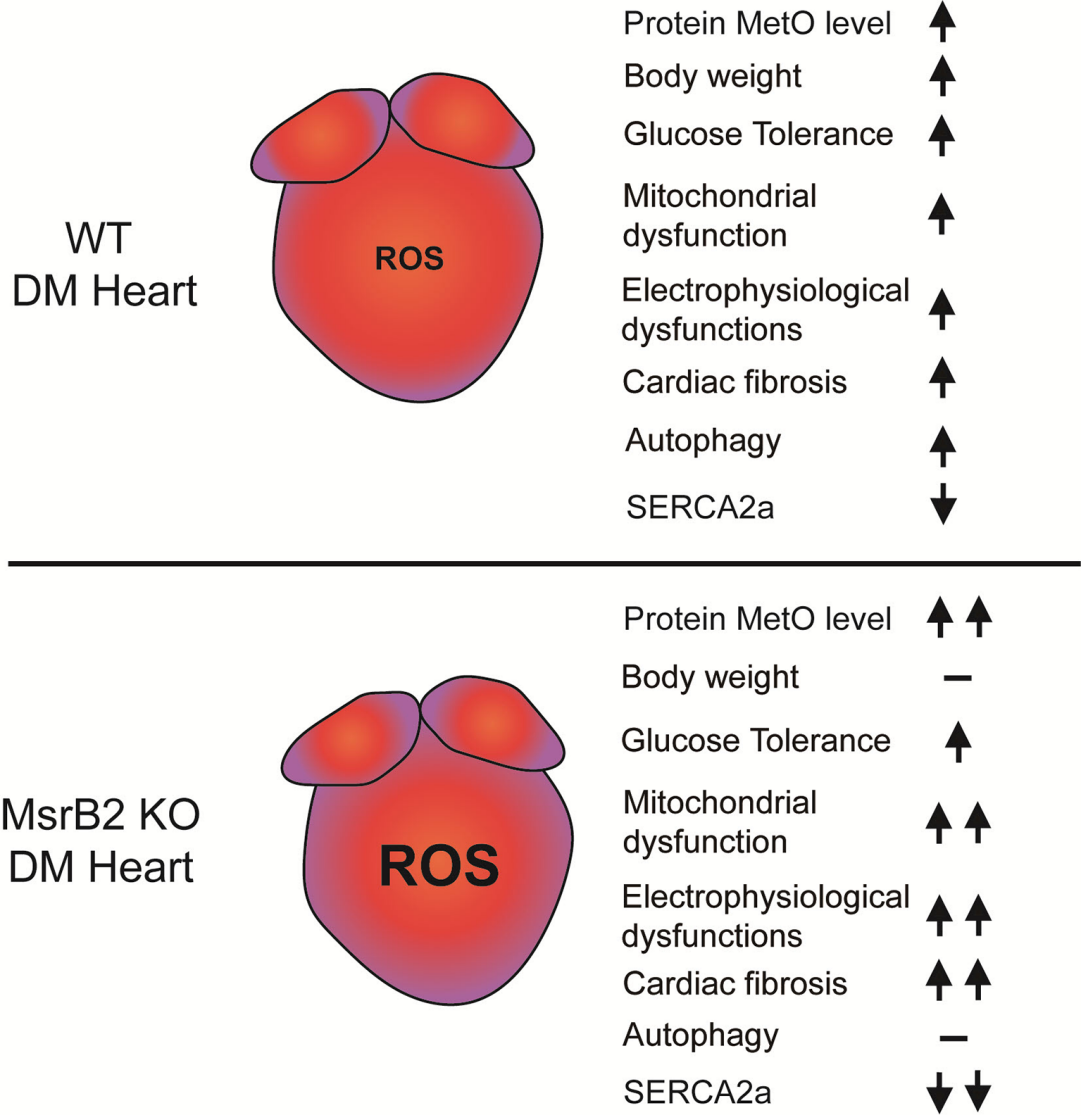

**Supplementary Information:**

The online version contains supplementary material available at 10.1186/s13098-024-01390-0.

## Background

Diabetes mellitus (DM) is a progressive and chronic metabolic disorder characterized by hyperglycemia due to impaired insulin levels, sensitivity, or action. In 2016, 14.4% (approximately 5.02 million) of Korean adults had diabetes [1]. In the US, over 28.5 million adults have been diagnosed with DM, while an estimated 8.5 million have undiagnosed DM and 96 million adults have prediabetes [2]. 65% of patients with DM will die from thrombotic cardiovascular events, including heart attack and stroke [3, 4]. Heart failure in DM (diabetic cardiomyopathy) increases with factors such as atherosclerosis, hypertension, and myocardial dysfunction [5, 6]. Myocardial stiffness through matrix protein accumulation (fibrosis) contributes to the development of diabetic cardiomyopathy (DCM) [7]. However, other pathophysiological mechanisms, such as myocardial insulin resistance, reactive oxygen species (ROS), and inflammation, are related to the development of DCM; conversely, mitophagy can improve cardiac dysfunction in DCM, but the precise molecular protective mechanism conferred by mitophagy in DCM is not fully understood [8]. DCM occurs by alteration of myofibrillar ATPase activity and sarcomeric reticulum functions induced by abnormal Ca^2+^ [9–12]. SERCA2a is important in DCM induction; moreover, SERCA2a overexpression improves mitochondrial quality control and attenuates cardiac microvascular ischemia-reperfusion injury [13].

Damaged mitochondria lead to highly increased ROS levels and further mitochondria damage in T1DM and T2DM. The increased ROS can change diverse protein functions through posttranslational modification such as carbonylation, 3-nitrotyrosine, s-sulfonation, s-nitrosylation, s-glutathionylation, disulfide formation [14, 15], methionine oxidation(MetO) [16, 17], and ubiquitination [18]. Further, ROS can increase mitophagy as a protective mechanism to remove damaged mitochondria and prevent excess production [13, 19, 20]. However, the molecular mechanism linking myocardial ROS to DCM remains unclear.

In diverse heart disease conditions, highly increased methionine oxidation has been identified in proteins such as thrombomodulin [16], calmodulin [21], and calcium–calmodulin-dependent protein kinase II [22]. Methionine oxidation by ROS requires reversal to restore the original protein function [23]. Methionine sulfoxide reductase (Msr) proteins [16, 24, 25] perform this task. Two types of Msr protein reduce methionine oxidation in mammals (MsrA and B). MsrA is localized to mitochondria, cytosol, and nucleus, MsrB1 to the cytosol and nucleus, and MsrB3 to the ER and mitochondria. MsrB2 is localized exclusively to the mitochondria [26–32] and highly expressed heart tissue [33]. MsrB2 is a well-identified mitophagy inducer in DM platelets [34]. In DM platelets, p53-dependent apoptosis is significantly increased by ROS, and JNK-dependent mitophagy is significantly activated as an inhibitory mechanism against ROS-induced apoptosis [35]. In this process, MsrB2 performs a major function in restoring Parkin and regulating damaged mitochondria to be regulated by mitophagy. However, the function of MsrB2 in other tissues in the context of diabetes is unknown.

We now provide evidence that MsrB2 expression is increased in DCM and MsrB2 plays a protective role in DCM by regulating mitophagy and mitochondrial energy metabolism. Such findings provide new therapeutic targets in preventing diabetic cardiomyopathy.

## Methods

### Human heart tissue

The Yale Human Investigation Committee (protocol# 1,005,006,865) approved all human studies. Each subject consented to all data and sample use. No studies were performed outside of what was approved.

### Cell culture

Primary cardiomyocyte cultures were prepared from C57Bl/6 WT and MsrB2 KO mice (Ehler et al., 2013). Briefly, the ventricular tissue was enzymatically dissociated, and the resulting cell suspension was enriched for cardiomyocytes by centrifugation. Then, isolated cardiomyocytes were plated onto collagen-coated culture dishes (Corning). To induce hypertrophy and diabetic cardiomyopathy, cardiomyocytes were cultured in a serum-free medium for ≥ 4 h and treated with 100 ng Endothelin-1 (ET-1) or 25 mM glucose for 48 h.

### Transient transfection

We purchased Parkin and MsrB2 ORF clones from OriGene (USA) and subcloned them into RFP- and GFP-tagged vectors, respectively. Cherry-LC3 was purchased by Addgene (#40,827). According to the manufacturer’s protocol, MsrB2-GFP, RFP-Pakin, and Cherry LC3 were transfected into H9C2 with Lipofectamine 3000 (Invitrogen, USA). Cells were harvested for 48 h in lysis buffer (50 mM Tris–HCl (pH 7.4), 150 mM NaCl, 0.25% Triton X-100, and a protease inhibitor cocktail) for further experiments.

### Preparation of diabetic mice

All mice had a C57Bl/6 background (WT and MsrB2 KO). For generating diabetic (DM) mice, 8-week-old mice were fed for 12 weeks a high-fat diet (HFD) after five days of STZ injection. The animals were housed at the Yale Animal Facility 300 George St. New Haven, CT, under the supervision of Yale Animal Resources Center and Rita Weber (Animal facility manager, Yale CVRC) or at the animal facility in Korea NIH (LML-KCDC-11-2-26). All experiments were performed under the appropriate guidelines and regulations under the approved protocols 2017–11,413 and KDCA-IACUC-22-041. The fact that the mice had the same genetic background and were often siblings meant that there was no significant variance within the groups. Any differences would, therefore, be directly related to treatment or modification. The experiments were corroborated using other mouse groups. Mice were randomly assigned to induction of DM groups. The experimenter was blinded to the blood glucose level. As described in the Results section, further experiment validation was performed using different approaches, i.e., chemical inhibition, chemical activation, and genetic knockout. We combined multiple randomized groups with multiple methods to reduce any bias.

### ECG recording

Anesthesia was provided through the induction chamber of a rodent respiratory anesthesia machine (3–4%). After anesthesia induction, an oral respiratory anesthesia device was connected to maintain the anesthesia (2%). After breathing stabilization, the mice were fixed in a ventrodorsal position, and the ECG (−) lead was connected to the left arm, the (+) lead to the right arm, and the background lead to the right leg. ECG recording was performed for 3 min (IX-BIO4). After recording, the lead was removed, and the subject was transferred to a cage to maintain body temperature.

### Glucose tolerance test (GTT)

WT and MsrB2 KO were fed an HFD for 12 weeks after STZ injection once per day for 5 days, then food and water starved for 24 h. After starvation, 1.5 g/mg glucose was injected intraperitoneally. To test glucose tolerance, checked glucose concentration was measured in a time dependent manner (0, 15, 30, 60, 90, 120 min).

### Western blotting

Heart tissue lysates were obtained by homogenization in a lysis buffer (50mM Tris-HCl [pH 7.4], 150mM NaCl, 0.25% Triton X-100, and protease inhibitor cocktail [Sigma-Aldrich]). of protein lysates were loaded in each well, and ≥ 3 independent replicates were used for quantification. We analyzed the band intensity using ImageJ analysis software (NIH) and converted the intensity value to fold change compared to HC or the non-treated group. Fold values were then used for statistical analysis.

### Electron microscopy

Samples of mouse heart tissue were fixed with 2% glutaraldehyde and 2% paraformaldehyde in 0.1 M sodium cacodylate (pH 7.4) for 2 h at room temperature (Fig. 2). They were washed three times with 0.1 M cacodylate buffer at room temperature, and cells were postfixed with 1% osmium tetroxide in 0.1 M cacodylate buffer for 1 h at room temperature. After rinsing with cold distilled water, tissue samples were dehydrated slowly with ethanol and propylene oxide. The samples were embedded in Embed-812 (EMS, USA) and visualized using a scanning electron microscope (Yale Biological EM Facility, New Haven, CT).

For Fig. 4 transmission electron microscopy (EM), the heart tissue was prefixed by incubating in 2% paraformaldehyde and 2.5% glutaraldehyde in 0.1 M phosphate buffer (pH 7.4) to prevent autolysis. To minimize the chemical reaction between pre- and post-fixation, the grids were washed thrice using the same buffer as in the fixative solution and postfixed with 1% osmium tetroxide. After washing thrice with deionized water, the samples were dehydrated with an ascending series of 30%, 50%, 70%, 80%, 90%, and 100% ethanol, followed by exchange for propylene oxide. Next, the tissues were embedded in Epon812 plastic resin and polymerized at 65ºC for 48 h. The prepared specimen was then cut into 70-nm thin sections using an ultramicrotome (EM-UC7, LEICA); the sections were mounted on a 100-mesh copper grid and electro-stained with 4% uranyl acetate. The section was observed with a transmission electron microscope (Libra120, Carl Zeiss, Germany) at an acceleration voltage of 120 kV.

### RNA sequencing

Total RNA was isolated from WT and MsrB2 mouse hearts (nonDM and DM). Bionics, Korea, performed RNA sequencing.

### ROS measurement

H9C2 cells were allowed to settle on glass-bottom dishes and treated with 5, 25 mM glucose or ET-1 for 48 h (Sigma-Aldrich). The treated cells were incubated with 1 µM H2DCFDA for 1 h and observed using an Invitrogen EVOS M5000 Cell Imaging System (Invitrogen) with a 20× lens. The signal intensity was calculated using the ImageJ program.

### Immunoprecipitation

Mouse heart tissue lysates and cell lysates (after transient transfection) were mixed with the specific target antibody [1 µg of LC3 anti-rabbit antibody (Abcam) and 2 µg of Parkin anti-goat antibody (Abcam) or 1.5 µg of Parkin anti-rabbit antibody (Abcam) for Parkin IP; 1 µg of MsrB2 anti-rabbit antibody (Yale) for MsrB2 IP; and GFP-Trap bead (Chromotek, USA), and the same species IgG control with HC] and incubated overnight at 4 °C. Then, 50% slurry protein A sepharose bead and 50% slurry protein G sepharose bead were mixed 50:50 and 30 µL of the 50% slurry washed A/G bead with lysates/antibody mixture was incubated for 1 h at 4 °C. After three further washes with lysis buffer, we used 1–10% lysates as input.

### Immunocytochemistry and confocal microscopy

H9C2 cells were allowed to settle on glass-bottom dishes and transiently transfected using MsrB2-GFP, RFP-Parkin, and Cherry-LC3. Then, the cells were fixed with a 4% paraformaldehyde solution (biosesang, Korea) and observed using a Nikon Eclipse-Ti confocal microscope with a 100x oil immersion lens. Colocalization was assessed using the parameters set in the Volocity software (PerkinElmer, USA).

### Statistics

Mouse studies were blinded to glucose levels. Where appropriate, all data are expressed as mean ± standard deviation or mean ± standard error of the mean. The nonparametric *t*-test was performed for comparisons between the two groups. One-way or two-way ANOVA analysis of variance was performed to compare four groups as outlined in the separate experiments. Analysis was performed with Prism software (GraphPad Software, Inc., La Jolla, CA). A *P* < 0.05 was considered to indicate statistically significant differences.

## Results

### ROS increases methionine sulfoxylation and methionine sulfoxide reductase (MsrB2) levels in DM mouse heart tissue

**Fig. 1:**
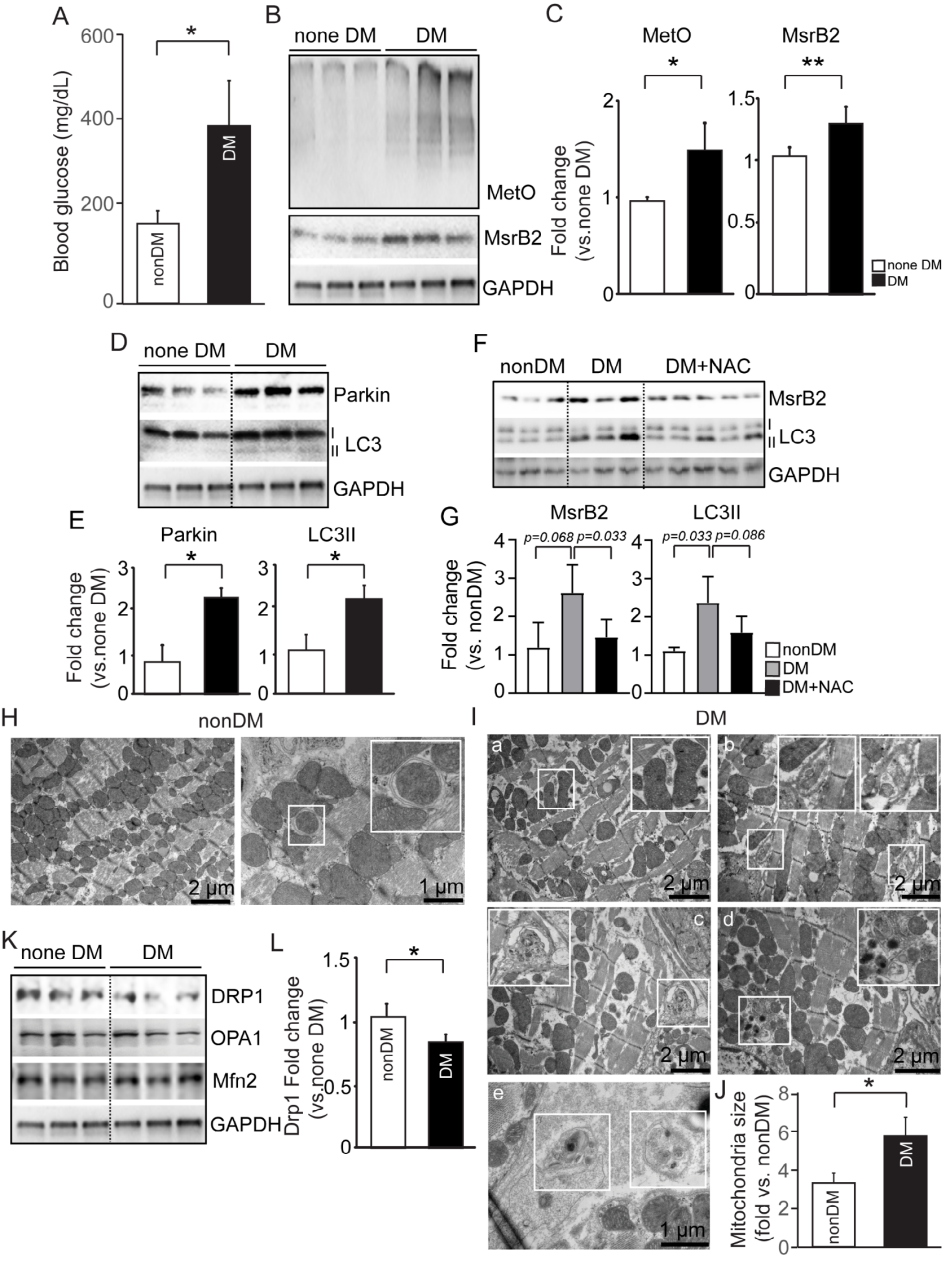
ROS induced methionine sulfoxidation (MetO) and mitochondrial dysfunction in the heart of diabetic mice. **A**. Blood Glucose in nonDM and DM mice. **B**. Western blot analysis of MetO and MsrB2 in nonDM (#1–3) and DM mouse hearts (DM #1–3). GAPDH served as a loading control. **C**. Quantification of MetO and MsrB2 signal intensity. **D**. Western blot analysis of Parkin and LC3II in nonDM (#1–3) and DM mouse hearts (DM #1–3). GAPDH served as a loading control. **E**. Quantification of Parkin and LC3II signal intensity. **F**. Western blot analysis of MsrB2 and LC3I/II in nonDM (#1–3), DM mouse hearts (DM #1–3), and DM with NAC treatment (#1–5). GAPDH served as a loading control. **G**. Quantification of MsrB2 and LC3II signal intensity. **H**. Electron microscopy (EM) in nonDM mice. The white body indicates a mitophagy structure. **I**. EM in DM mice. The White body indicates mitophagy (autophagosome and autolysosome) structure. **J**. Quantification of mitochondria size in nonDM and DM mice. **K**. Western blot analysis of DRP1 and DRP1 and Mfn2 in nonDM (#1–3) and DM mouse hearts (DM #1–3). GAPDH served as a loading control. **L**. Quantification of DRP1signal intensity. The nonparametric *t*-test was performed for *p values*

The DM mouse model was initially assessed for MsrB2 cardiac expression (5 days of STZ injection, followed by 12 weeks of HFD). The DM mice had glucose concentrations nearly twice those of nonDM mice (Fig. 1A). Methionine sulfoxylation (MetO) levels resulting from ROS and MsrB2 were both significantly increased in DM mice (MetO; normal heart (*n* = 3); 0.97 ± 0.03, DM heart (*n* = 3) 1.29 ± 0.12, *P* = 0.013 and MsrB2; normal heart (*n* = 3); 1.05 ± 0.06, DM heart (*n* = 3) 1.29 ± 0.05, *P* = 0.009) **(**Fig. 1B and C**).** MsrB2 has been reported to increase mitophagy (selective mitochondria autophagy) by binding to Parkin and LC3II in platelets of DM [34]. In mouse DM hearts, consistent with the MsrB2 increase, the levels of Parkin (normal heart (*n* = 3); 0.98 ± 0.37, DM heart (*n* = 3) 2.19 ± 0.39, *P* = 0.01) and the active form of LC3 also increased (normal heart (*n* = 3); 0.85 ± 0.28, DM heart (*n* = 3) 2.16 ± 0.38, *P* = 0.009) (Fig. 1D and E). Then, the ROS inhibitor, NAC, was intraperitoneally administered to DM mice to determine whether ROS regulates the increase in MsrB2 expression and mitophagy observed in DM heart tissue. The mice were sacrificed 1 h later. MsrB2 and LCII expression increased with ROS and were suppressed when NAC reduced ROS (Fig. 1F and G). In H9C2 cardiac myoblast cells, ROS and LC3 activation were highly increased by high glucose(HG) (Additional file 1) and hypertrophic condition by endothelin-1(ET-1) treatment (Additional file 2). In ET-1 treatment conditions, they highly increased MsrB2 with ROS and LC3 activation. These results support an association of MsrB2 and LC3 activation with ROS where cardiac mitophagy may be involved.

### Mitochondrial disrupted by DM mouse heart tissue

Mitochondrial structural integrity was assessed through EM (Fig. 1H and I). The myocardium was evenly distributed in the nonDM mouse heart tissue, and the mitochondria between the contractile machinery were closely aligned (Fig. 1H). In contrast, DM hearts showed an unevenly distributed myocardial structure with an irregular mitochondrial shape (either very large or very small). Interestingly, the mean total mitochondrial size was significantly increased in DM hearts compared to normal mice (Fig. 1I and J). Since such changes can be closely related to mitochondrial fission and fusion, DRP1 was used as a fission marker, and OPA1 and Mfn2 were used as fusion markers. We confirmed decreased DRP1 expression and no significant changes in OPA1 or Mfn2 expression (Fig. 1K and L). In EM images of the heart tissue from DM mice, vacuoles containing organelles could be observed (Fig. 1Ib–Ie). These structures are typical autophagosomes or autolysosomes and support increased autophagy in the DM heart along with increased Parkin and the active form of LC3II. When such structures contain damaged mitochondria, this further supports an increased mitophagy autophagosome (Fig. 1Ib). Thus, our results support that DM mouse hearts had increased ROS (increased MetO) levels and MsrB2 induction and mitophagy on a background of likely impaired mitochondrial function and mitochondrial biogenesis.

### MsrB2, a modulator of DM mouse cardiac complications

Autophagy is activated in the hearts of DM mice, as shown in Figs. 1 and 4, demonstrating that the high blood glucose and ROS production induced by DM leads to mitochondrial dysfunction and increased levels of MsrB2 and mitophagy in cardiac tissue. However, there are no reports of MsrB2 function in the DM heart. In this study, an MsrB2 global KO (MsrB2 KO) was generated to investigate the role of MsrB2 and the increase in mitophagy in DM heart disease. This mouse, first described by Lee et al. [34], has a complete absence of MsrB2 expression in tissues such as the liver, heart, and aorta (Additional file 3). In a chronic, bodily disease such as diabetes, a global knockout better reflects the natural effects of a germline mutation or variant in the MsrB2 gene that a patient might experience.

**Fig. 2:**
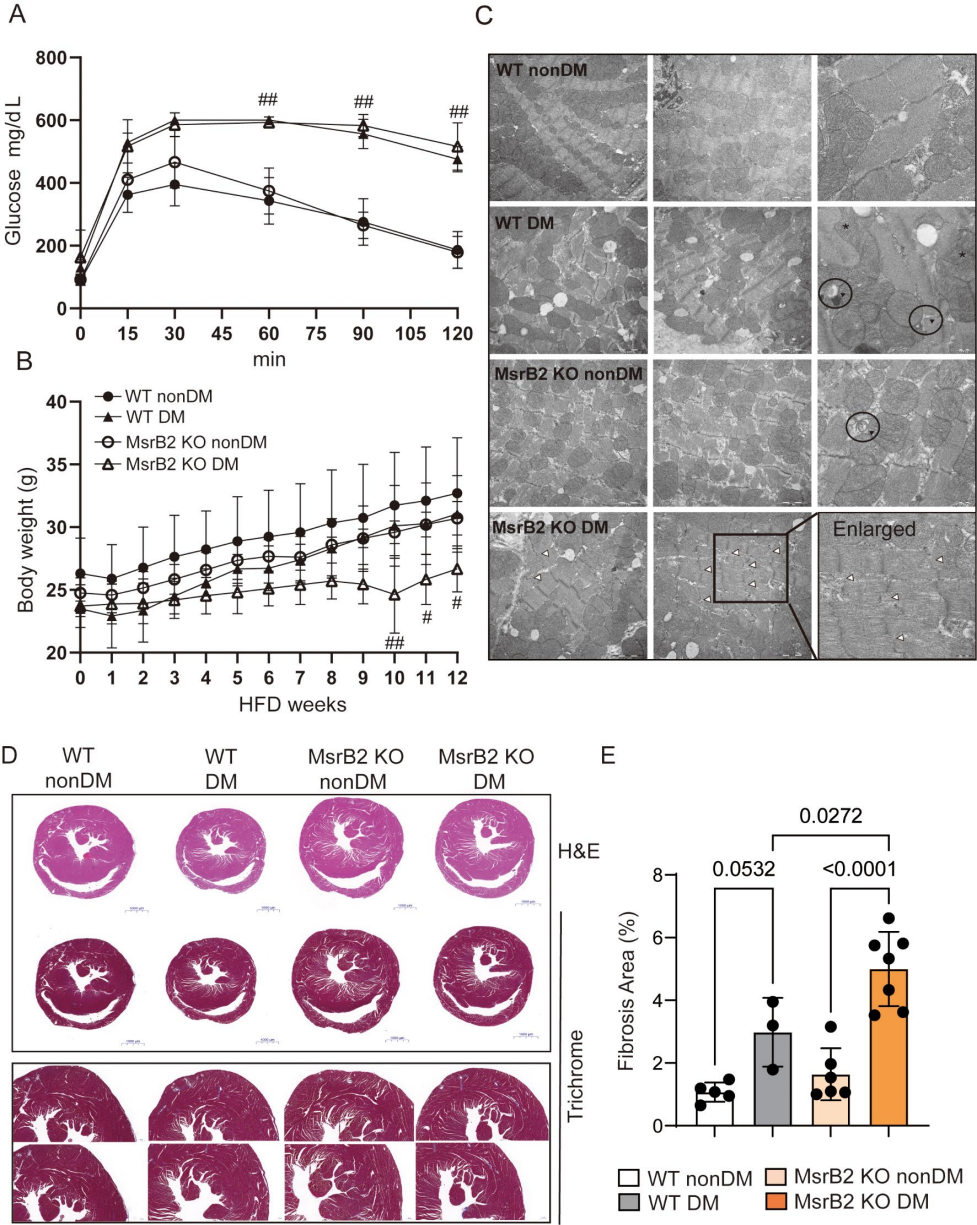
Cardiac complications occur in MsrB2 KO DM. **A**. Glucose tolerance test (GTT) in WT and MsrB2 KO under nonDM and DM conditions. **B**. Body weight in WT and MsrB2 KO DM mice during HFD feeding. The body weight was weighed every week. **C**. Electron microscopy (EM) images in WT and MsrB2 KO DM mice. The white arrow indicates the mitochondria (autophagosome and autolysosome). The black triangle indicates the membrane structure. A white triangle indicates an abnormal Z-line. **D**. H&E and trichrome staining of histological sections of the heart of WT and MsrB2 KO mice under nonDM and DM MsrB2 conditions. **E**. Fibrotic areas were quantified using the ImageJ (FIJI) analyzer on histological sections. The two-way ANOVA analysis was performed for *p values*

To confirm the role of MsrB2 in the heart complications caused by diabetes, MsrB2 KO mice received HFD for 12 weeks at 5 days after STZ injection to create a DM mouse model. A glucose tolerance test (GTT) confirmed that the KO did not cause differences in GTT between DM and WT mice (Fig. 2A**)**. The body weight decreased in MsrB2 KO DM mice compared with WT DM and nonDM mice (Fig. 2B). In EM images of heart tissue from WT DM and MsrB2 KO DM mice, mitochondrial irregularities were observed in both conditions (Fig. 2C, asterisk) as well as membrane structures like elongated autophagosome membranes (Fig. 2C, black triangles). Notably, irregular patterns (broken and uneven) in the Z-line of the sarcomere structure were observed only in MsrB2 KO DM mice (Fig. 2C, white triangle). Histological analysis of heart tissue also revealed significantly increased cardiac fibrosis (Fig. 2D and E), hemorrhage Additional file 4, yellow triangle), tissue fragmentation (Additional file 4, circle), and large vacuoles (Additional file 4, green triangle) in MsrB2 KO DM mice compared to WT DM mice.

**Fig. 3:**
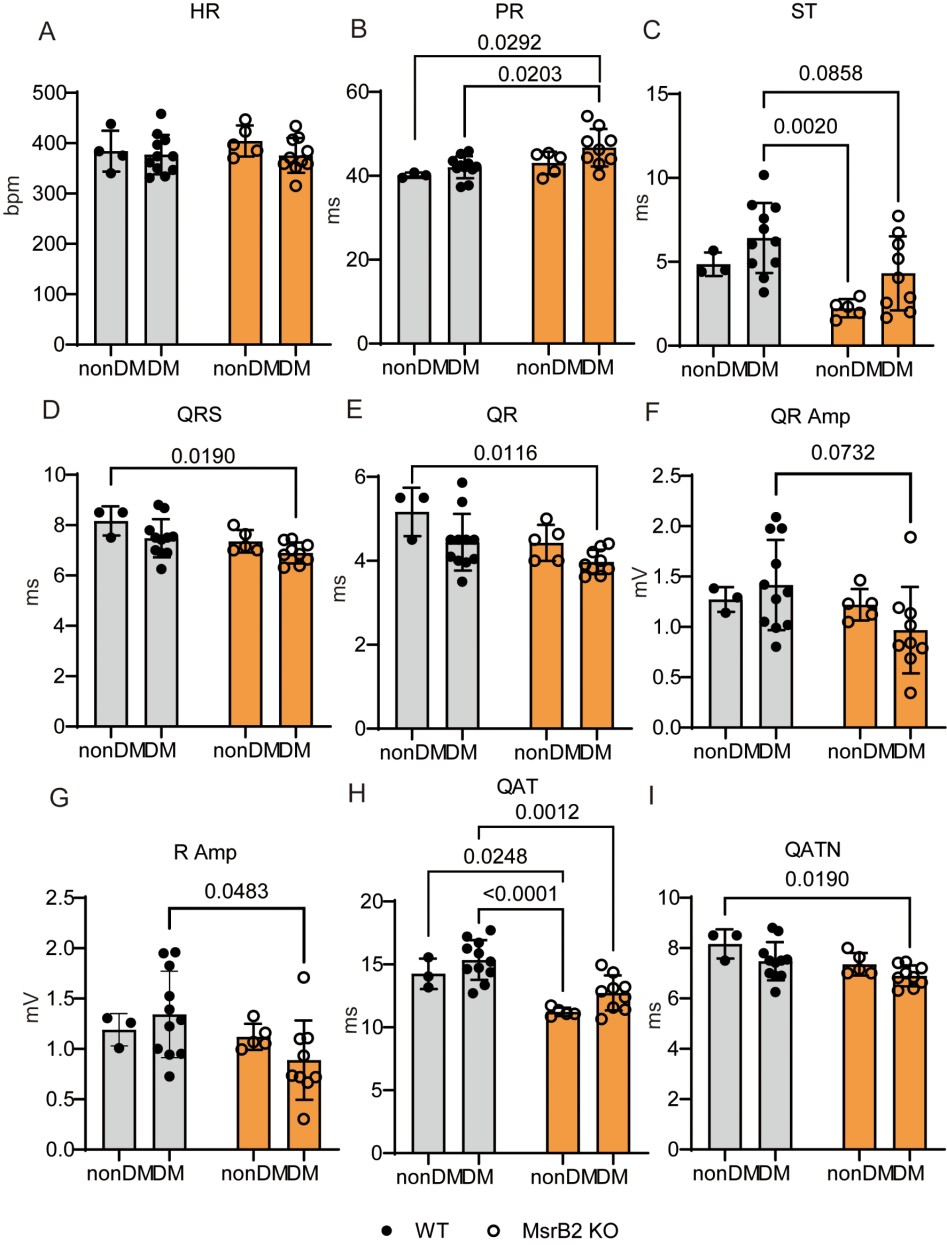
Electrophysiological dysfunctions occurred in MsrB2 KO DM mice. **A** ~ **I**. ECG recording results in nonDM (WT #1–4, MsrB2 KO #1–5) and DM (WT #1–11, MsrB2. KO #1–9) mouse hearts

Heart rate did not differ in all groups (Fig. 3A). Still, we observed an increased PR interval(PR interval on an ECG shows how long it takes for the heart’s electrical signals to travel from the top of the heart to the bottom of the heart) in MsrB2 KO DM compared with WT DM (Fig. 3B), suggesting that these results may be caused by atrial depolarization dysfunction in MsrB2 KO DM. ECG parameters such as ST, QRS, QR, QR amplitude, and R amplitude were reduced (Fig. 3C-G), and these findings indicate impaired ventricular depolarization function in MsrB2 KO DM. Additionally, we confirmed that abnormal ventricular repolarization resulted from decreased QAT and QATN (Fig. 3H and I). These ECG results demonstrate abnormal electrophysiological functions in MsrB2 KO DM.

**Fig. 4:**
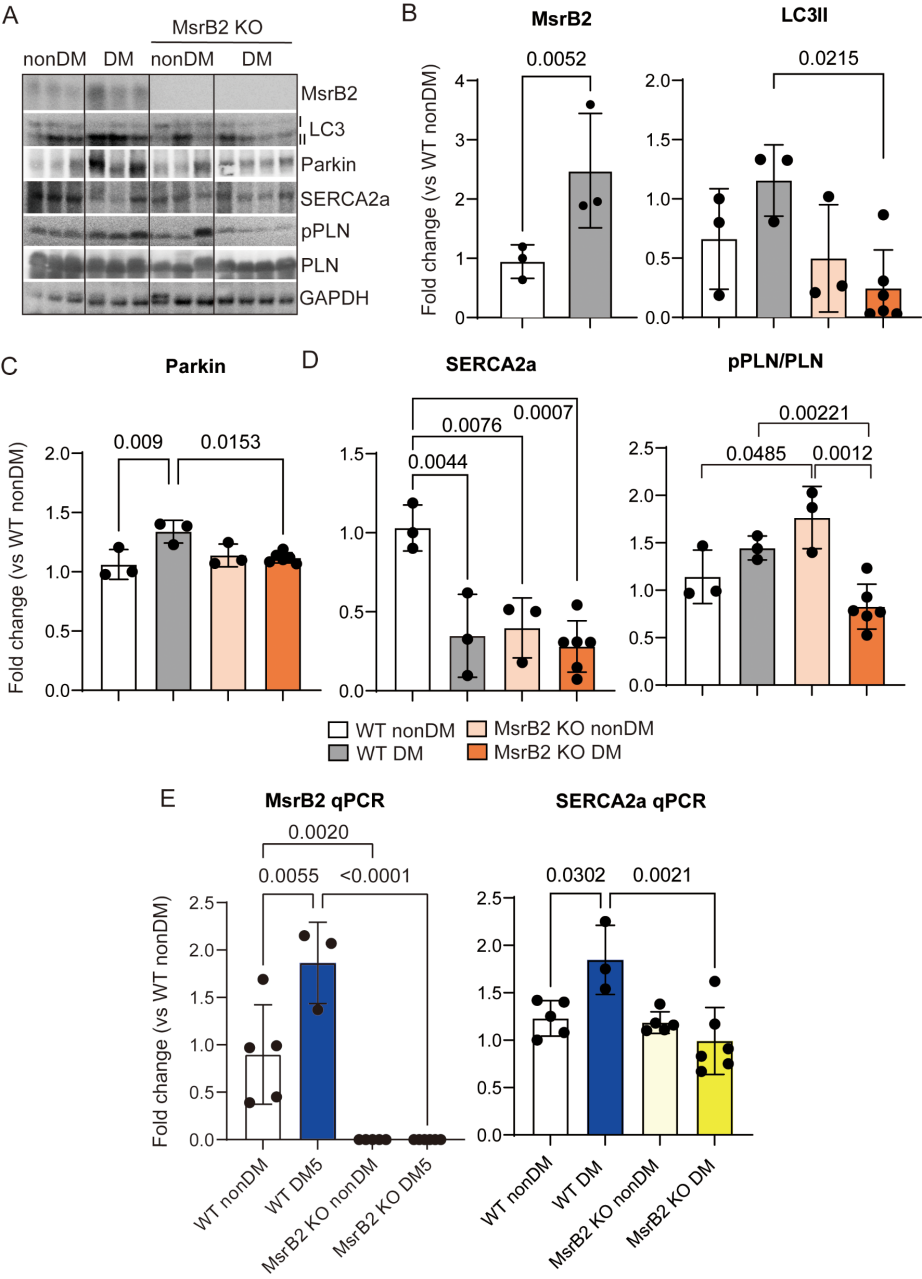
Decreased autophagy and SERCA2a-PLN function in MsrB2 KO DM mice. **A**. A Western blot analysis of MsrB2, LC3I/II, Parkin, SERCA2a, pPNL, PNL, and GAPDH of nonDM (WT #1–3, MsrB2 KO #1–3) and DM mouse (WT #1–3, MsrB2 KO #1–6) hearts. GAPDH served as a loading control. **B**–**D**. Quantification of MsrB2, LC3I/II, Parkin, SERCA2a, pPNL/PNL, and GAPDH signal intensity. **E**. Quantitative RT-PCR analysis MsrB2 and SERCA2a transcript levels in nonDM (WT #1–5, MsrB2 KO #1–5) and DM (WT #1–3, MsrB2 KO #1–6) mouse hearts. The two-way ANOVA analysis was performed for *p values*

Further, MsrB2 and LC3II expression was increased in the DM hearts (Fig. 4A and B), but the active form of LC3 (LC3II) was not increased in MsrB2 KO nonDM or MsrB2 KO DM mice (Fig. 4A and B). These results highlight the pivotal role of MsrB2 as a critical activator of autophagy in the diabetic heart. In MsrB2 KO DM, cardiac muscle structures were compromised (Fig. 3) and a marked increase in cardiac fibrosis was noted (Fig. 2). Therefore, we confirmed the expression of SERCA2a as a marker of DCM. Previous reports have highlighted that DCM occurs due to alterations in SERCA2a function induced by abnormal Ca^2+^ levels. Moreover, SERCA2a expression levels are closely associated with mitochondrial quality control and mitigation of cardiac microvascular ischemia-reperfusion injury [13]. ROS is excessively increased in cardiac lipid overload conditions, causing arrhythmia and sudden death by sarcomeric reticulum calcium leakage. The expression of SERCA2a was significantly decreased in WT DM, MsrB2 KO nonDM, and MsrB2 KO DM mice, but phosphorylated phospholamban (PLN) was significantly reduced only in the MsrB2 KO DM (Fig. 4A and D). Additionally, as observed by qPCR, SERCA2a mRNA levels were decreased in MsrB2 KO DM mice (Fig. 4E).

To validate changes in mitochondrial function in MsrB2 KO DM, mRNA sequencing was performed. The sequencing data revealed upregulated expression of oxidative phosphorylation (OXPHOS)-related genes and genes involved in ROS production. This finding was consistent with the sequencing results and qPCR analysis (Additional file 5D). However, the total mitochondrial content, specifically mitochondrial DNA (mtDNA), remained unchanged (Additional file 5D). These results indicate that there is excessive ROS accumulation in MsrB2 KO DM hearts from mice due to heightened OXPHOS and ROS production. Furthermore, the mRNA sequencing data from the hearts of MsrB2 KO DM mice revealed elevated expression of Bdh1 and Acat1, which are involved in ketone degradation (Additional file 5B).Consequently, this ROS accumulation contributes to overall cardiac dysfunction on a global scale. Metabolic abnormalities occurred in MsrB2 KO DM hearts compared with WT DM hearts. Both glucose and fatty acid metabolism were highly decreased in the MsrB2 KO DM (Additional file 6).

Notably, despite the similar glucose tolerance of WT DM and MsrB2 KO DM mice, mitochondrial dysfunction, cardiac fibrosis, myocardial tissue abnormalities, and decreased body weight were observed in MsrB2 KO DM mice.

### MsrB2 is a factor inducing autophagy in hyperglycemic conditions

**Fig. 5:**
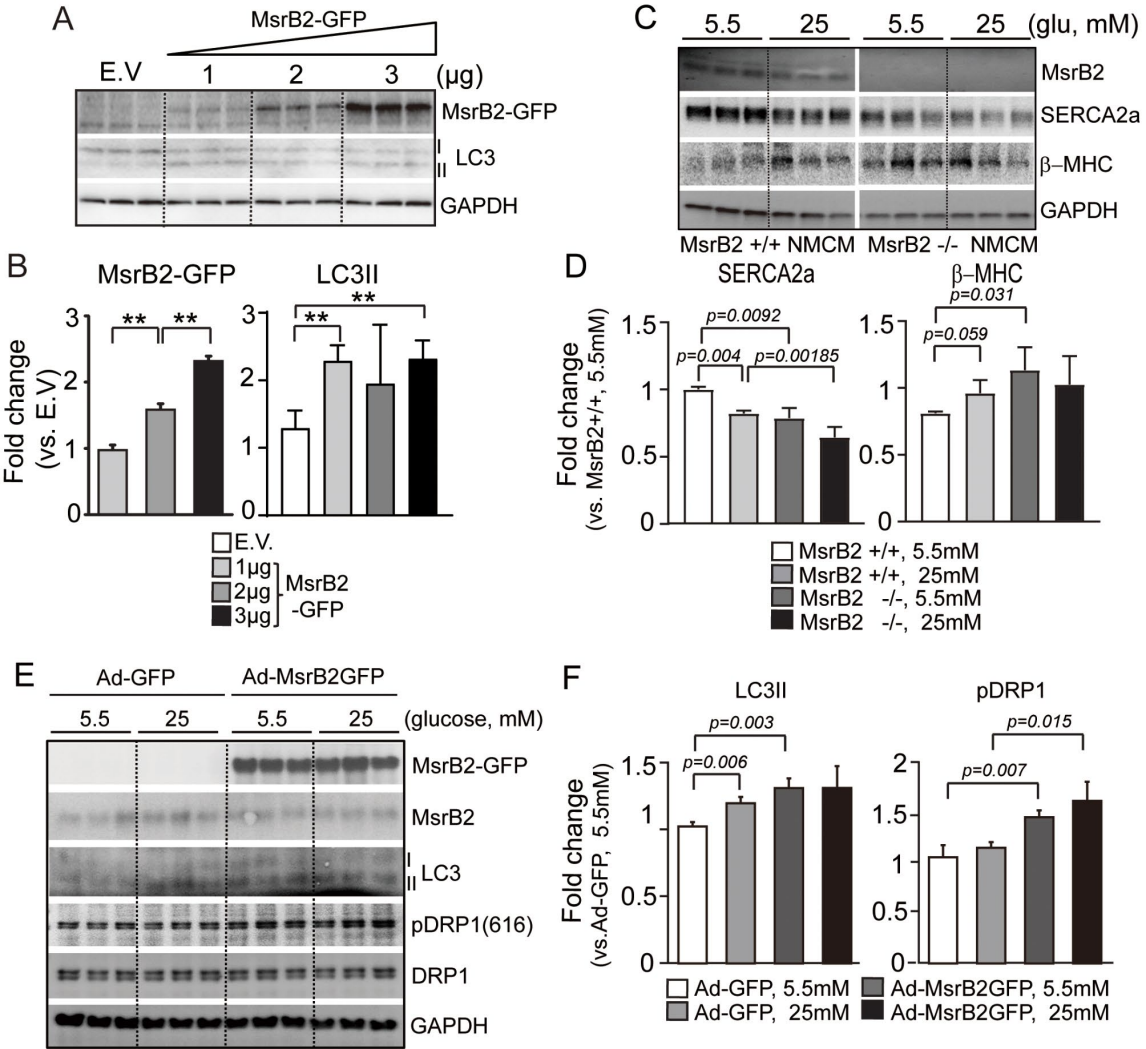
MsrB2 induces autophagy by LC3 activation. **A**. Western blot analysis of MsrB2 and LC3I/II in H9C2 after MsrB2-GFP transfection. MsrB2 induced LC3II in a concentration-dependent manner. GAPDH served as a loading control. **B**. Quantification of MsrB2 and LC3II signal intensity. **C**. Western blot analysis of MsrB2, SERCA2a, and bMHC in WT and MsrB2 KO NMCM. After 4 h of starvation, 5.5 or 25 mM glucose was treated for 48 h. GAPDH served as a loading control. **D**. Quantification SERCA2a and bMHC signal intensity. **E**. Western blot analysis of MsrB2, LC3I/II, pDRP1, and DRP1 in NMCM after Ad-MsrB2-GFP infection. Ad-MsrB2-GFP 50 m.o.i infected into NMCM. Then, 5.5 or 25 mM glucose was treated for 48 h after 4 h starvation. GAPDH served as a loading control. **F**. Quantification of LC3II and pDRP1 signal intensity. The one-way ANOVA analysis was performed for *p values*

Consistent with the mouse model results, HG concentrations induce ROS and increase autophagy activity (LC3II induction) in H9C2 cells (Additional file 1). Additionally, ET-1 (a myocardial hypertrophy inducer) induced ROS and increased autophagy and MsrB2 expression in H9C2 cells (Additional file 2). Autophagy increased in an MsrB2 concentration-dependent manner (Fig. 5A and B). When MNCM was isolated from WT mice and treated with 25 mM glucose, SERCA2a expression decreased (Fig. 5C and D). In MsrB2 KO MNCM, expression of b-MHC increased in WT and MsrB2 KO MNCM while that of SERCA2a significantly decreased in MsrB2 KO cells treated with 25 mM glucose compared to WT MNCM treated with 25 mM glucose (Fig. 5C and D). When MsrB2 expression was increased using an adenovirus capable of overexpressing MsrB2 in mouse neonatal cardiomyocytes (MNCM), autophagy and DRP1 activity increased (Fig. 5E and F). These results support that MsrB2 depletion induced a heart failure phenotype in MNCM.

We then assessed for direct interactions between MsrB2 and Parkin. No interaction between MsrB2 and Parkin was observed in nonDM; however, we observed binding between MsrB2 and Parkin in DM mouse hearts (Additional file 7 A). To ensure binding between Parkin, MsrB2, and LC3II in cells, MsrB2-GFP, RFP-Parkin, and Cherry-LC3 plasmids were used. The binding between each factor was confirmed through immunoprecipitation (Additional file 7B and 7 C), and immunostaining was performed to verify colocalization between the three intracellular proteins (Additional file 7D). When MsrB2-GFP, RFP-Parkin, and Cherry-LC3 plasmids were overexpressed in H9C2 cells treated with the mitophagy inducer CCCP, LC3 signals co-localized with MsrB2 (Additional file 7D). In addition, we confirmed the colocalization of MsrB2 and Parkin in mitochondria (Additional file 7D). Parkin accumulated around the mitochondria during CCCP treatment (Additional file 7D).

### Elevation of MetO in human DM heart tissue

**Fig. 6:**
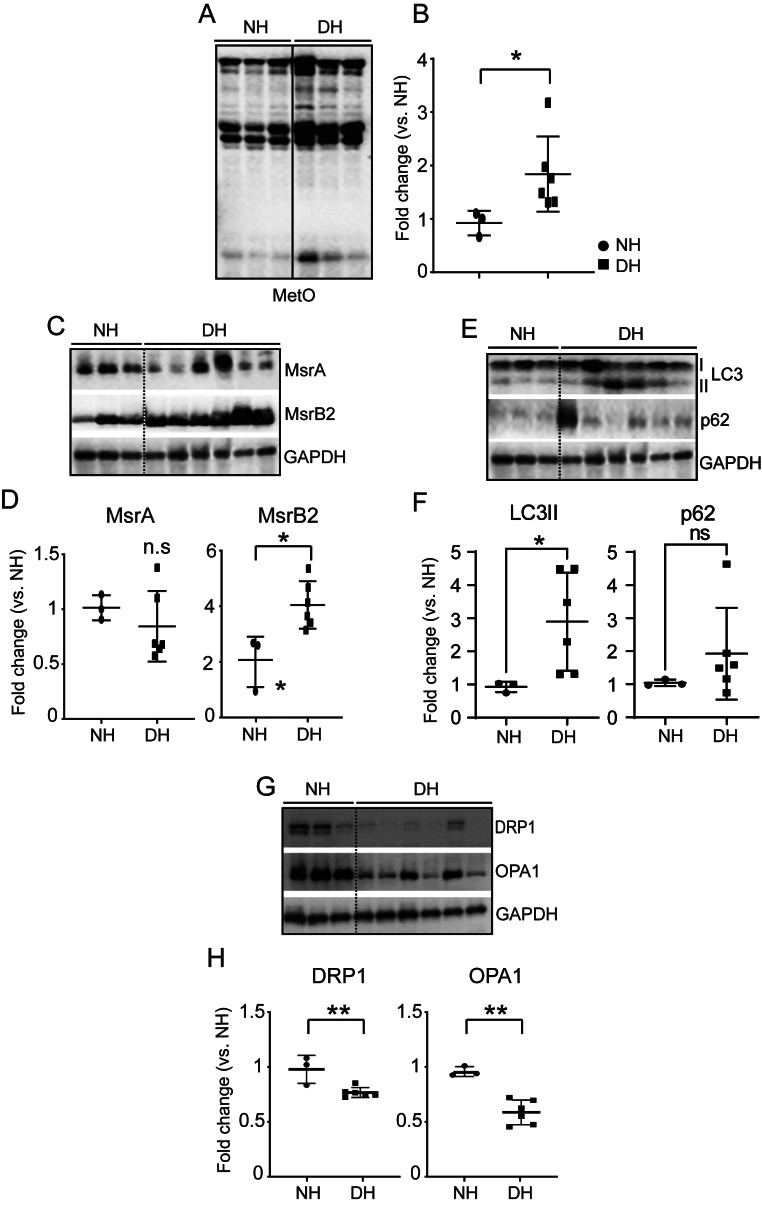
ROS induced methionine sulfoxidation (MetO) in human diabetic hearts. **A**. Western blot analysis of MetO in normal (NH #1–3) and diabetic human heart tissue(DH #1–6). GAPDH served as a loading control. **B**. Quantification of MetO signal intensity. **C**. Western blot analysis of methionine sulfoxide reductase A and B2 (MsrA and B2) in normal (NH #1–3) and diabetic heart tissue (DH #1–6). GAPDH served as loading a loading control. **D**. Quantification of MsrA and MsrB2 signal intensity. **E**. Western blot analysis of LC3l/II and p62 in normal (NH #1–3) and diabetic heart tissue (DH #1–6). GAPDH served as a loading control. **F**. Quantification of LC3l/II and p62 signal intensity. **G**. Western blot analysis of DRP1 and OPA1 in normal (NH #1–3) and diabetic heart tissues (DH #1–6). GAPDH served as a loading control. **H**. Quantification of MetO intensity

In the human DM heart, there was increased MetO compared with the normal heart (normal heart (*n* = 3); 0.92 ± 0.23, DM heart (*n* = 6) 1.83 ± 0.70, *P* = 0.02) **(**Fig. 6A and B**).** We then determined MsrA and B expression levels in human DM (HD) hearts (MsrA and MsrB1–B3). The expression of all Msr proteins was not significantly changed compared to the human normal (HN) heart (Fig. 6C and D). LC3 II levels and Parkin expression significantly increased but those of p62 did not change substantially compared to HN hearts (Fig. 6E and F, (Additional file 8). We found evidence of mitochondrial dysfunction by analyzing the expression of proteins involved in mitochondria regeneration, DRP1 and OPA1, in DM heart tissue **(**Fig. 6G and H**)**. The expression of DRP1 and OPA1 was significantly reduced in HD hearts compared to HN hearts, suggesting defective mitochondrial biogenesis in HD hearts. We concluded that excessive ROS (MetO indicates excessive ROS) and inactivation of autophagy in the HD heart interfere with normal biological functions, inhibiting mitochondrial biogenesis and leading to severe mitochondrial dysfunction. MsrB2 directly regulates the activity of LC3 and its interaction with Parkin and LC3II. Thus, the autophagic/mitophagy process activated by MsrB2 may be cytoprotective during diabetes.

**Fig. 7:**
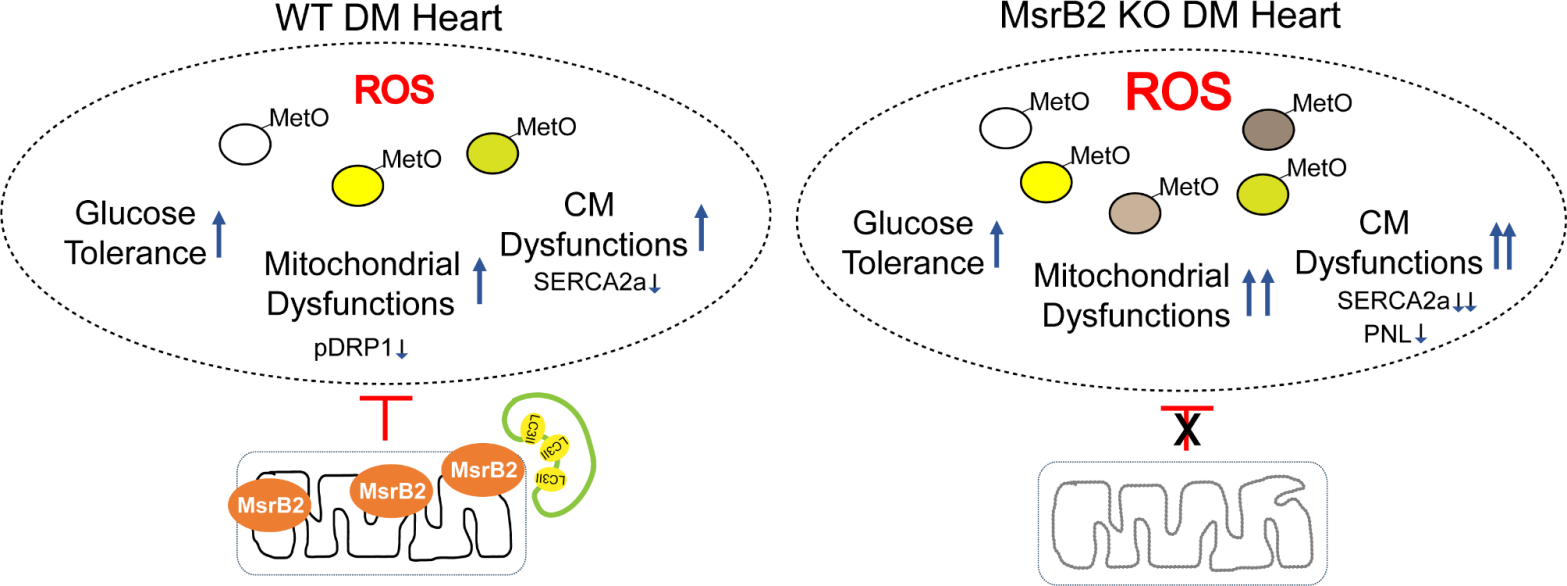
Summary. Increased MsrB2 expression in diabetic mouse hearts suppressed cardiac complications. These results were confirmed by inducing diabetes in mice with suppressed MsrB2 expression (MsrB2 KO). In MsrB2 KO DM, the expression of SERCA2a, which regulates myocardial contractility, was reduced, cardiac fibrosis increased, and the number of mitochondrial structural abnormalities increased

In DM hearts, protein MetO is increased by ROS. ROS leads to mitochondrial and myocardial dysfunction. MsrB2, which ROS induces, plays a role in inhibiting such oxidative damage through LC3 activation and direct interaction with Parkin and LC3II, leading to autophagy induction, particularly mitophagy. The absence of MsrB2 exacerbates mitochondrial and myocardial damage, resulting in severe cardiac dysfunction in DM. These findings are summarized in Fig. 7.

## Discussion

In our mouse model, we observed increased MsrB2 only in DM hearts compared to DM muscle, liver, or brain (Additional file 3). Concurrently, the biosynthesis of mitochondria (involving mitochondrial fission and fusion) was significantly reduced in both human and mouse DM hearts due to excessive ROS; however, only the mouse DM heart showed activation of autophagy. Interestingly, heart tissues from DM patient autopsies exhibited a more severe DM phenotype than the mouse model used in this study. The mouse DM heart was characterized by MsrB2 induced autophagy, leading to partially preserved mitochondrial structure and function. Excessive ROS dramatically reduced mitochondrial biosynthesis (mitochondrial fission and fusion) in human and mouse DM hearts. Accordingly, we concluded that MsrB2 expression is increased in the DM heart, having a protective function not reported previously. Subsequently, we confirmed that MsrB2 plays a critical protective role in the DM heart by regulating mitochondria and energy metabolism and inducing mitophagy. Nonetheless, the protective effect of mitophagy remains controversial, as some studies have reported their protective effects while others have reported the induction of fibrosis [36–43]. In our research, MsrB2 was found to directly regulate the activity of LC3 to initiate the autophagy process (Fig. 5), and it interacted with Parkin similarly to that in platelets (Additional file 7). In MsrB2 KO DM, it was confirmed that decreased expression of MsrB2 led to inactivation of LC3II and parkin expression (Fig. 4), mitochondrial dysfunctions (Fig. 2), and inhibition of energy metabolism (Additional file 8) due to excessive ROS production (Additional file 7). As well as MsrB2 regulated SERCA2a and phospholamban activity (Figs. 4 and 5). Ultimately, all the dysfunctions in MsrB2 KO DM reduce myocardial function and induce diabetic complications. However, we need further studies related to autophagy flux and induction of cardiac complications by MsrB2.

This may be explained by excessive chronic stress, where excess mitophagy may be detrimental. Our model supports an initial protective effect but does not exclude autophagy/mitophagy as an inducer of apoptosis, likely dependent upon the severity and duration of DM.

Nearly all ATP in the normal adult mammalian heart is produced by mitochondrial oxidative phosphorylation [8, 44]. Fatty acid oxidation provides over 60% of the energy the heart consumes [8, 45]. RNA-seq analysis in WT DM and MsrB2 KO DM mice showed decreased glucose and increased fatty acid and ketone body metabolism (Additional file 6). However, the activity of both the TCA cycle regulator PDHa1 and the fatty acid oxidation regulator HADHA was decreased in MsrB2 KO DM mice, which is indirect evidence that energy supply was reduced in MsrB2 KO DM. It is then predicted to induce increased utilization of ketone bodies and NRF2-dependent OXPHOS-related gene expression in MsrB2 KO DM to overcome energy supply shortages. The ROS scavengers Gpx, Maoa, and catalase were decreased in MsrB2 KO DM, indicating excessive accumulation of ROS in the MsrB2 KO DM heart. All these results confirm that the imbalance of energy metabolism in diabetes leads to cardiac complications. Previous studies reported that ROS is excessively increased in cardiac lipid overload conditions, causing arrhythmia and sudden death by sarcomeric reticulum calcium leakage [46]. The finding that SERCA2a expression is decreased in MsrB2 KO DM supports the fact that regulating mitochondria function by MsrB2 is a major factor in controlling the development of DM heart complications. In addition, MsrB2 KO DM mice demonstrated lipid accumulation, cardiac fibrosis, hemorrhage, abnormalities in cardiac tissue, and electrophysiological dysfunctions compared to WT DM mice. In particular, the atrial depolarization time is shortened, but the ventricular depolarization and ventricular repolarization times are prolonged, which is a poor prognosis that can lead to arrhythmia [47, 48]. These are potential indicators of future heart failure and poor prognosis. There is increased MsrB2 expression observed in diabetes, and its decrease can induce more heart complications than WT DM. Therefore, measuring MsrB2 expression levels may predict cardiac complications from DM. MsrB2 KO DM mice do not appear to have better glucose tolerance, but aberrant mitochondrial metabolic activity can result in excessive ROS accumulation and subsequent weight loss. The characteristics of DM observed in MsrB2 KO DM mice are similar to those observed in lean diabetes, which is prevalent in the Asian population [49, 50].

Our results suggest that both WT DM and MsrB2 KO DM mice display similar patterns, indicating that MsrB2 is not a direct regulator of diabetes. These findings suggest that regulating MsrB2 expression to maintain mitochondrial energy metabolism balance may be an effective treatment strategy to prevent heart complications specific to Asian lean diabetes, such as those found in the Korean population. However, further research is required to fully understand the underlying mechanisms and validate the therapeutic potential of MsrB2 modulation in DM individuals.

In conclusion, our study suggests that MsrB2 is essential in regulating mitochondrial function and maintaining cardiac health in DM. Decreased expression is associated with cardiac complications, including fibrosis, hemorrhage, and tissue abnormalities. Therefore, measuring MsrB2 expression levels may be a valuable biomarker for predicting the likelihood of cardiac complications in DM patients. Additionally, the use of MsrB2 analogs or inducers may be a potential therapeutic strategy for managing DM heart disease. However, further studies are needed to validate these findings and explore the potential clinical applications of MsrB2 inducers in managing cardiovascular disease in DM patients.

### Electronic supplementary material

Below is the link to the electronic supplementary material.

Supplementary Material 1

## Data Availability

No datasets were generated or analysed during the current study.

## Abbreviations

DM: Diabetes Mellitus
GTT: Glucose Tolerance Test
HFD: High-Fat Diet
ROS: Reactive Oxygen Species
DCM: Diabetic Cardiomyopathy
MetO: Methionine Sulfoxylation
MsrA: Methionine Sulfoxide Reductase A
MsrB: Methionine Sulfoxide Reductase B
OXPHOS: Oxidative Phosphorylation
PLN: Phospholamban
STZ: Streptozotocin
TCA: Tricarboxylic Acid Cycle
CCCP: Carbonyl Cyanide 3-chlorophenylhydrazone
HADHA: Hydroxyacyl-CoA Dehydrogenase Trifunctional Multienzyme Complex Subunit Alpha
MNCM: Mouse Neonatal Cardiomyocyte
NAC: *N*-acetylcysteine
