# Supplementary material for: Methionine sulfoxide reductase B2 protects against cardiac complications in diabetes mellitus
Source: Diabetol Metab Syndr. 2024 Jul 5;16:149. doi: 10.1186/s13098-024-01390-0 (PMC11225187; doi:10.1186/s13098-024-01390-0)
Supplement: Supplementary file 1 — Supplementary Material 1 [file 13098_2024_1390_MOESM1_ESM.docx]

**Additional information**

**Material Methods.**

**Measurement of ROS**

H9C2 cells were allowed to settle on glass-bottom dishes and treated 5, 25mM glucose for 48hrs or ET-1(Sigma-Aldrich). The treated cells were incubated with 1uM H2DCFDA for 1hr and observed using an Invitrogen EVOS M5000 Cell Imaging System (Invitrogen) with a 20X lens. The signal intensity was calculated using the ImageJ program ()

**Additional File Legends**

**Additional File 1. ROS and autophagy activation increased in H9C2 cells due to high glucose conditions.**

1. Western blot analysis of LC31/II in H9C2 under 5.5 and 25mM glucose treated condition.
2. The H9C2 cells were incubated with 1uM H2DCFDA for 1hr and observed.
3. Quantification of green fluorescence signal intensity (n=3).

**Additional File 2. ROS and autophagy activation increased in H9C2 cells under ET-1 treatment.**

1. The H9C2 cells were incubated with 1uM H2DCFDA for 1hr after ET-1 treatment and observed.
2. Quantification of green fluorescence signal intensity (n=3).
3. Western blot analysis of MsrB2 and LC31/II in H9C2 under 5.5 and 25mM glucose treated condition.
4. Quantification of MsrB2 and LC3II signal intensity (n=3).

**Additional File 3. Tissue distribution of MsrB2 in Mice.**

1. Western blot analysis of MsrB2 in mice heart, muscle, liver and brain under nonDM and DM. GAPDH served as a loading control.
2. Western blot analysis of MsrB2 in WT and KO mice hearts, aorta and liver. Actin served as a loading control.

**Additional File 4. Cardiac tissue dysfunction occurs in MsrB2 KO DM mice.**

A. H&E of histological sections of the heart of WT and MsrB2 KO mice under nonDM and DM MsrB2 conditions.

B. Enlarged abnormal parts in WT DM and MsrB2 KO DM in H&E staining.

The two-way ANOVA analysis was performed for *p values*.

**Additional File 5. OXPHOS-related gene expression increased in MsrB2 KO DM mice.**

- 1. OXPHOS and ROS generation.
  2. 1.3-fold up and down regulated genes in MsrB2 KO DM compared with WT DM mice. Genes are categorized by mouse specific GO (Fatty acid metabolism, glucose metabolism, fatty acid oxidation, and cellular ketone metabolic process).
  3. 1.3-fold up and down regulated genes in MsrB2 KO DM compared with WT DM mice. Genes are categorized by mouse specific GO (OXPHOS and ROS biosynthetic process).
  4. Quantitative RT-PCR analysis ROS inducer (NOX4 and SOD1), OXPHOS (mND1) and mitochondria contents (mtDNA) transcript levels in nonDM (WT #1–5, MsrB2 KO #1–5) and DM (WT #1–3, MsrB2 KO #1–6) mouse hearts.

The two-way ANOVA analysis was performed for *p values*.

**Additional File 6. Metabolic dysfunctions occur in MsrB2 KO DM mice.**

1. Western blot analysis of HADHA, pPDHa1(S293), PDHa1, NRF2 and GAPDH of mice nonDM (WT #1–3, MsrB2 KO #1–3) and DM (WT #1–3, MsrB2 KO #1–6) hearts. GAPDH served as a loading control.

B~D. Quantification of pPDHa1/PDHa1, PDHa1, HADHA and NRF2 signal intensity.

**Additional File 7. MsrB2 induces mitophagy by interacting with Parkin and LC3II.**

1. Western blot analysis of Parkin, MsrB2, and GAPDH in mouse DM hearts. After immunoprecipitation with MsrB2 specific antibody, the Parkin signal was detected by Parkin-specific antibodies. GAPDH served as a loading control.
2. MsrB2-GFP with RFP-Parkin and MsrB2-RFP with GFP-Parkin were overexpressed in HEK293 cells by transient transfection. Immunoprecipitation with GFP-tagged beads, then MsrB2 or Parkin signal detected by specific antibodies.
3. The MsrB2-GFP with Cherry-LC3 was overexpressed in HEK293 cells by transient transfection. Immunoprecipitation with GFP-tagged beads, MsrB2 or LC3 signal detected by specific antibodies.
4. Confocal microscopy images using MsrB2-GFP, GFP-LC3, Cherry-LC3, RFP-Parkin, with or without CCCP in H9C2 myoblasts.
5. Western blot analysis of MsrB2 and LC3I/II in NMCM after MsrB2-GFP transfection. GAPDH served as loading control.

**Additional File 8. Mitophagy related PINK and Parkin expression in Human diabetic Heart.**

1. Western blot analysis of PINK and Parkin of human heart tissues in normal (NH #1–3) and diabetic heart (DH #1–6). GAPDH served as a loading control.
2. Quantification of PINK and Parkin signal intensity.

**Additional File 1
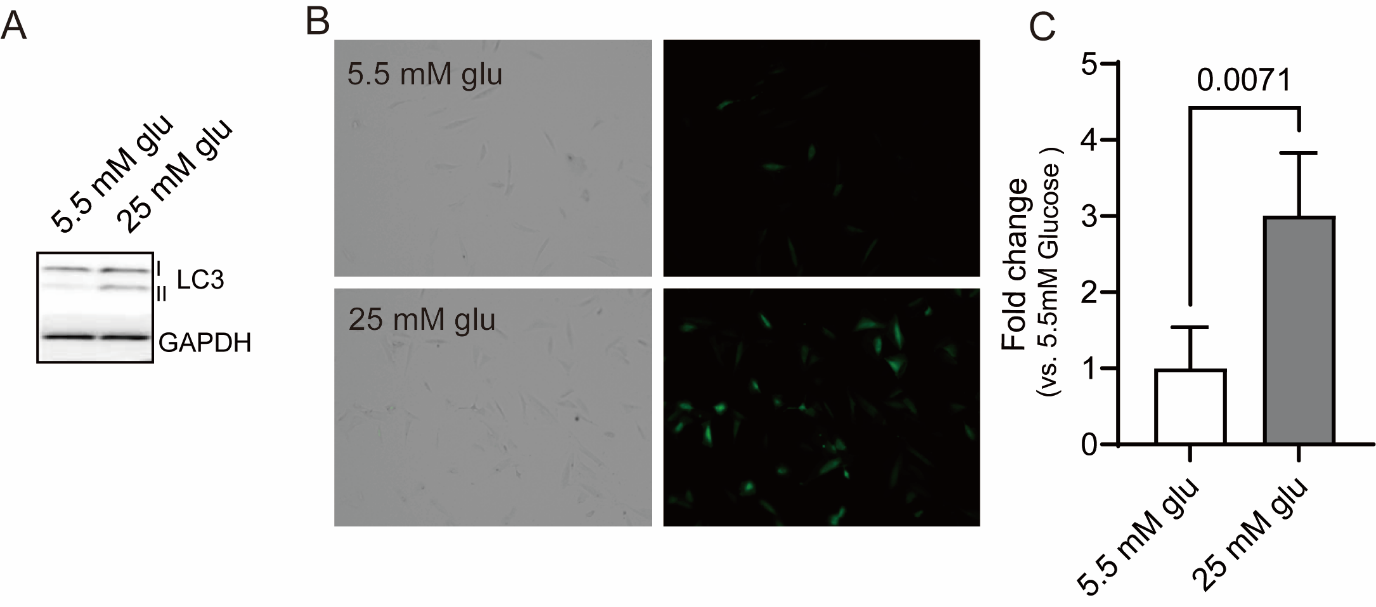
**

**Additional File 2**

**
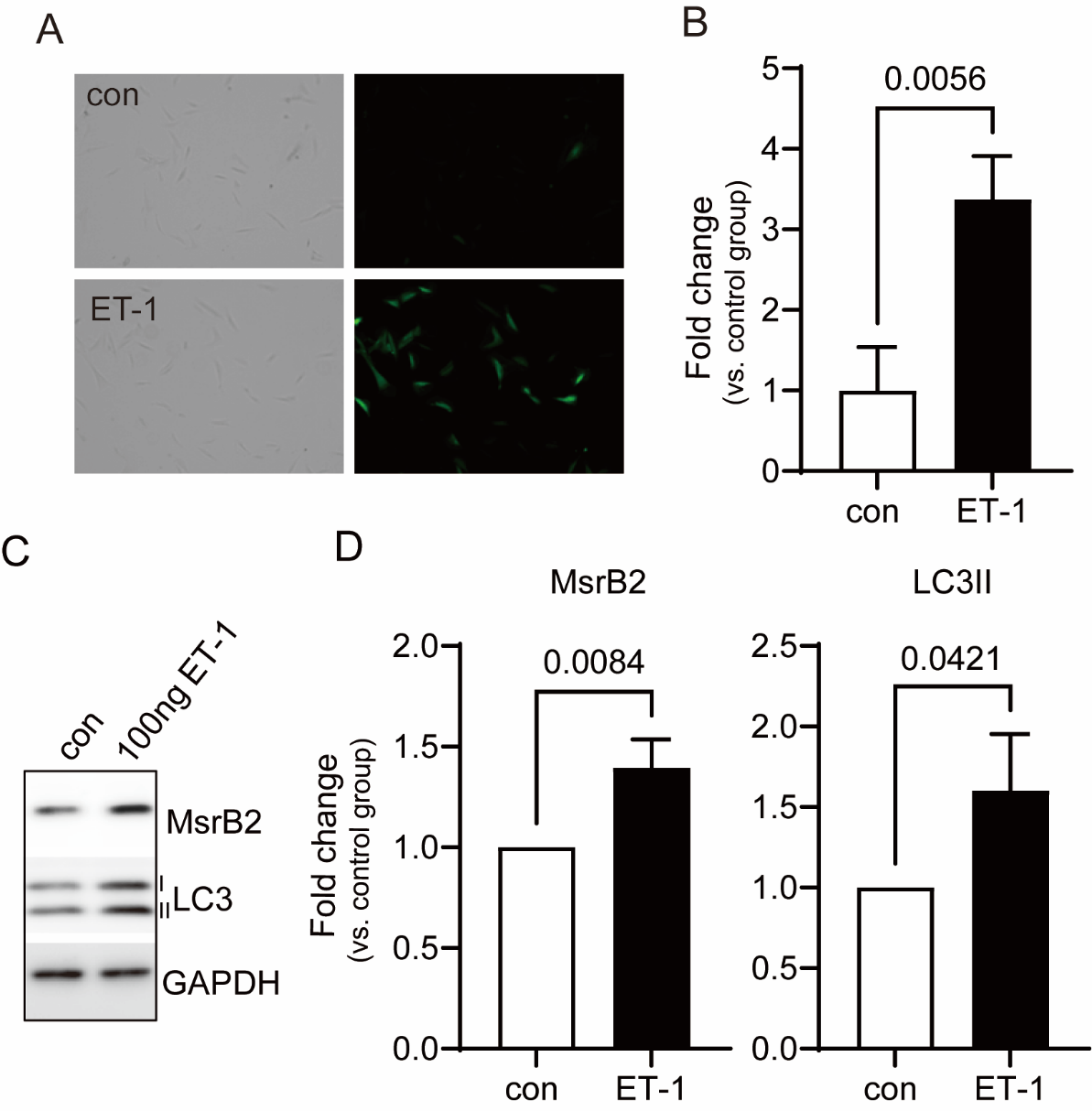
**

**Additional File 3**


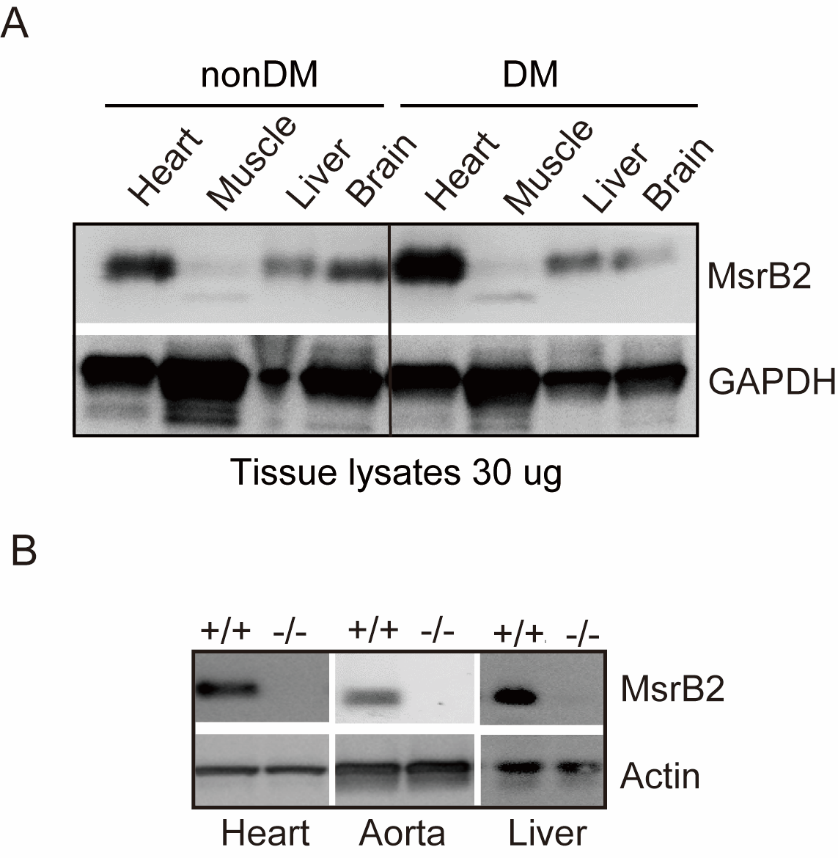


**Additional File 4**

**
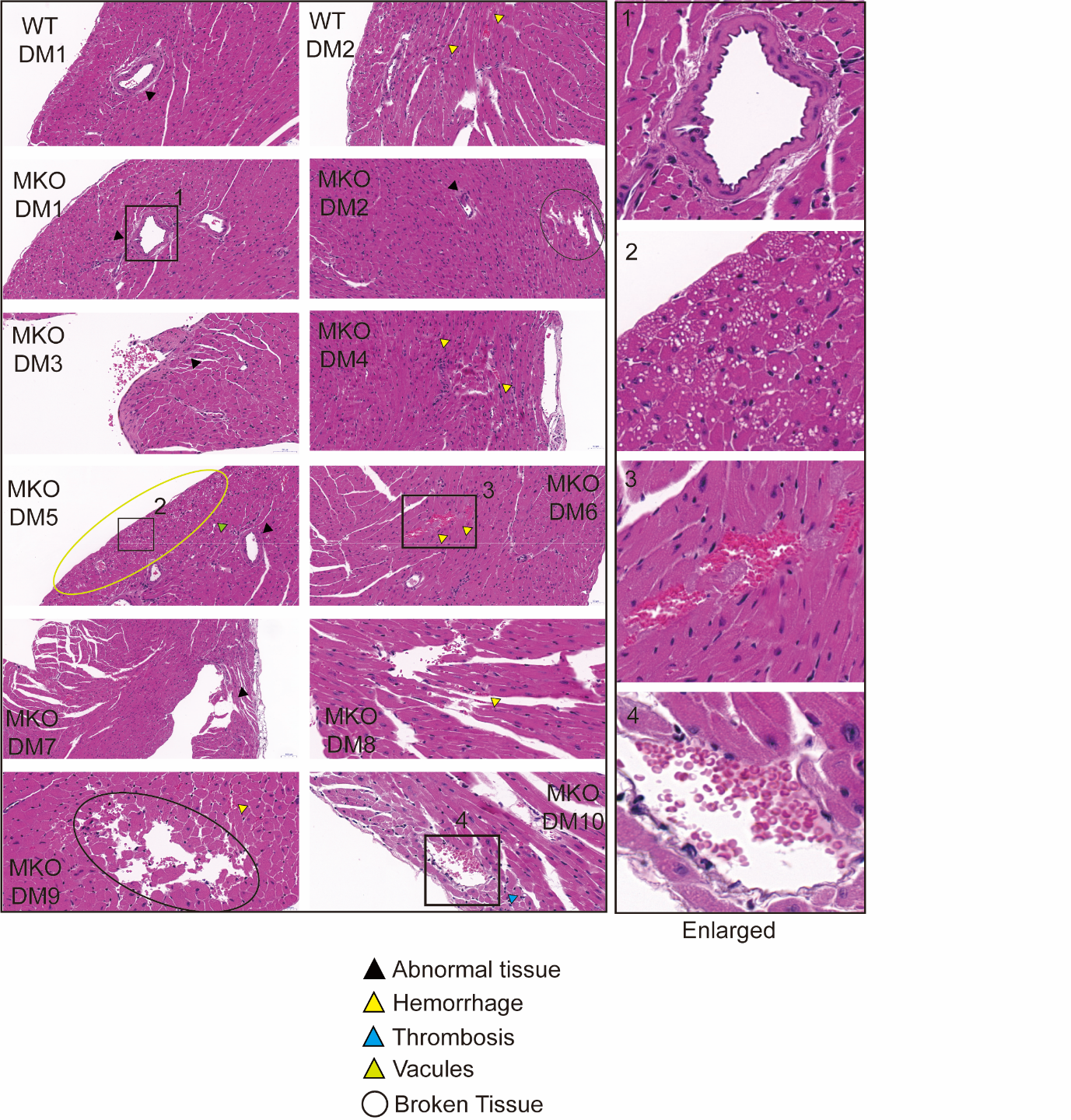
**

**Additional File 5**

**
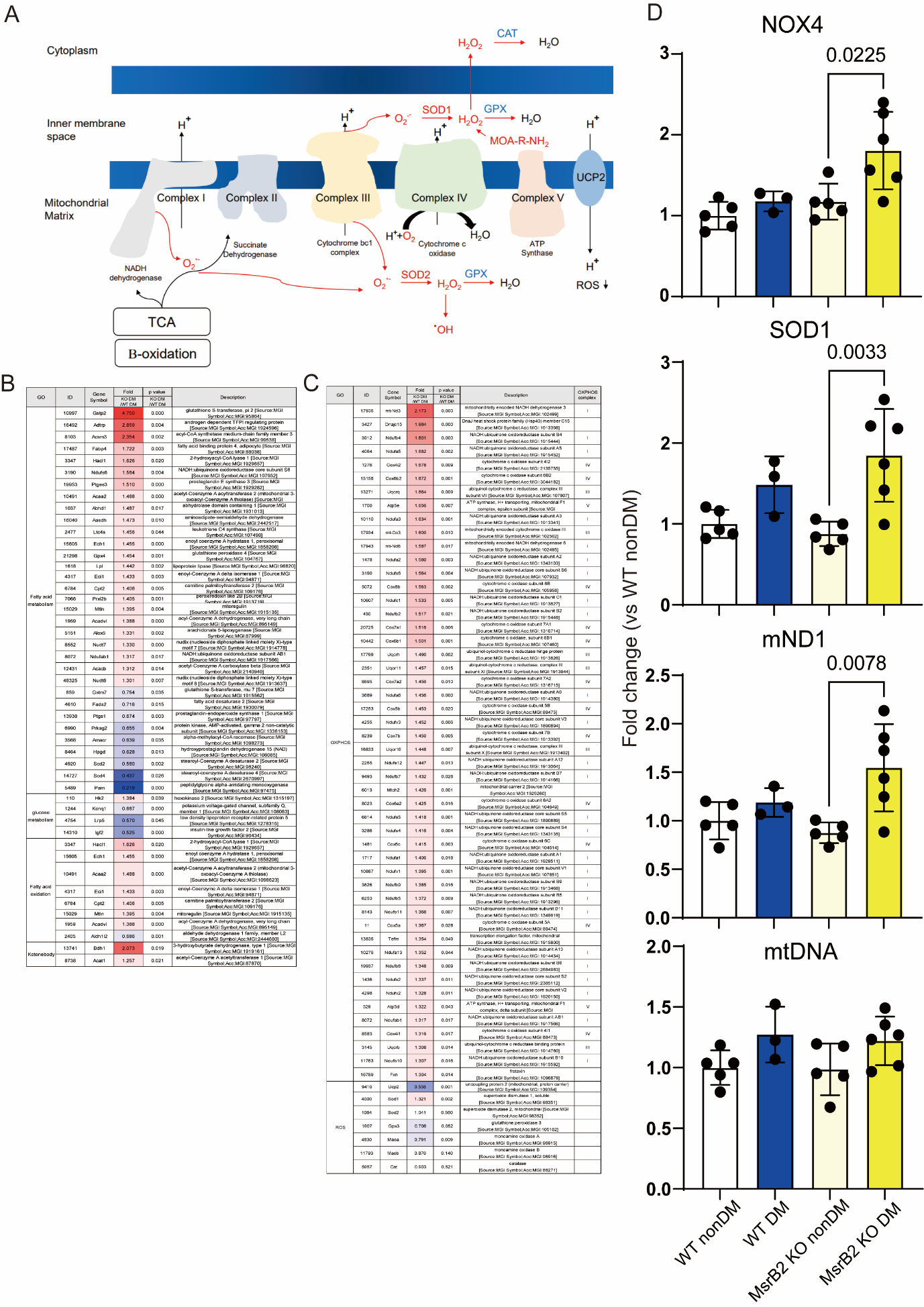
**

**Additional File 6**

**
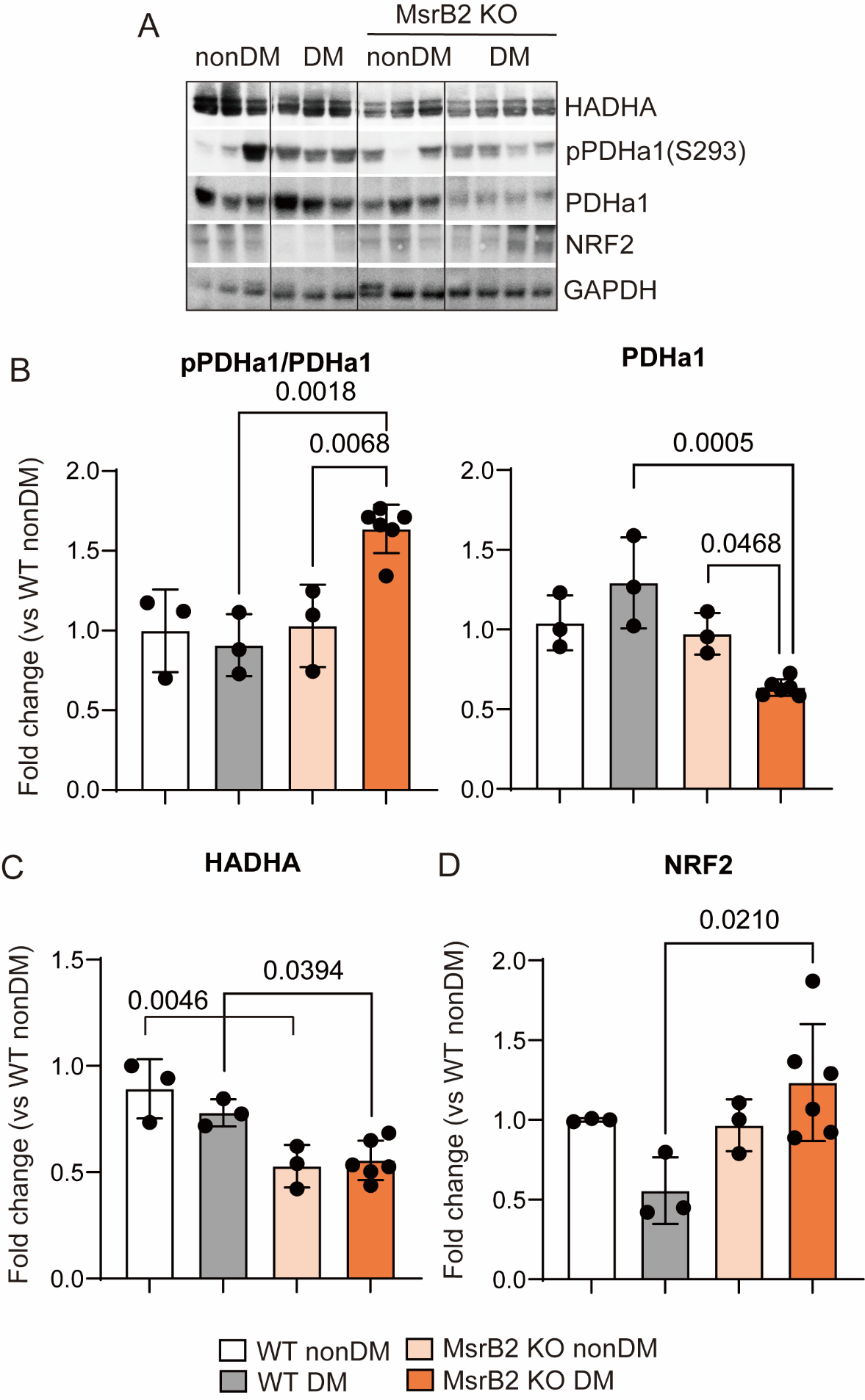
**

**Additional File 7**

**
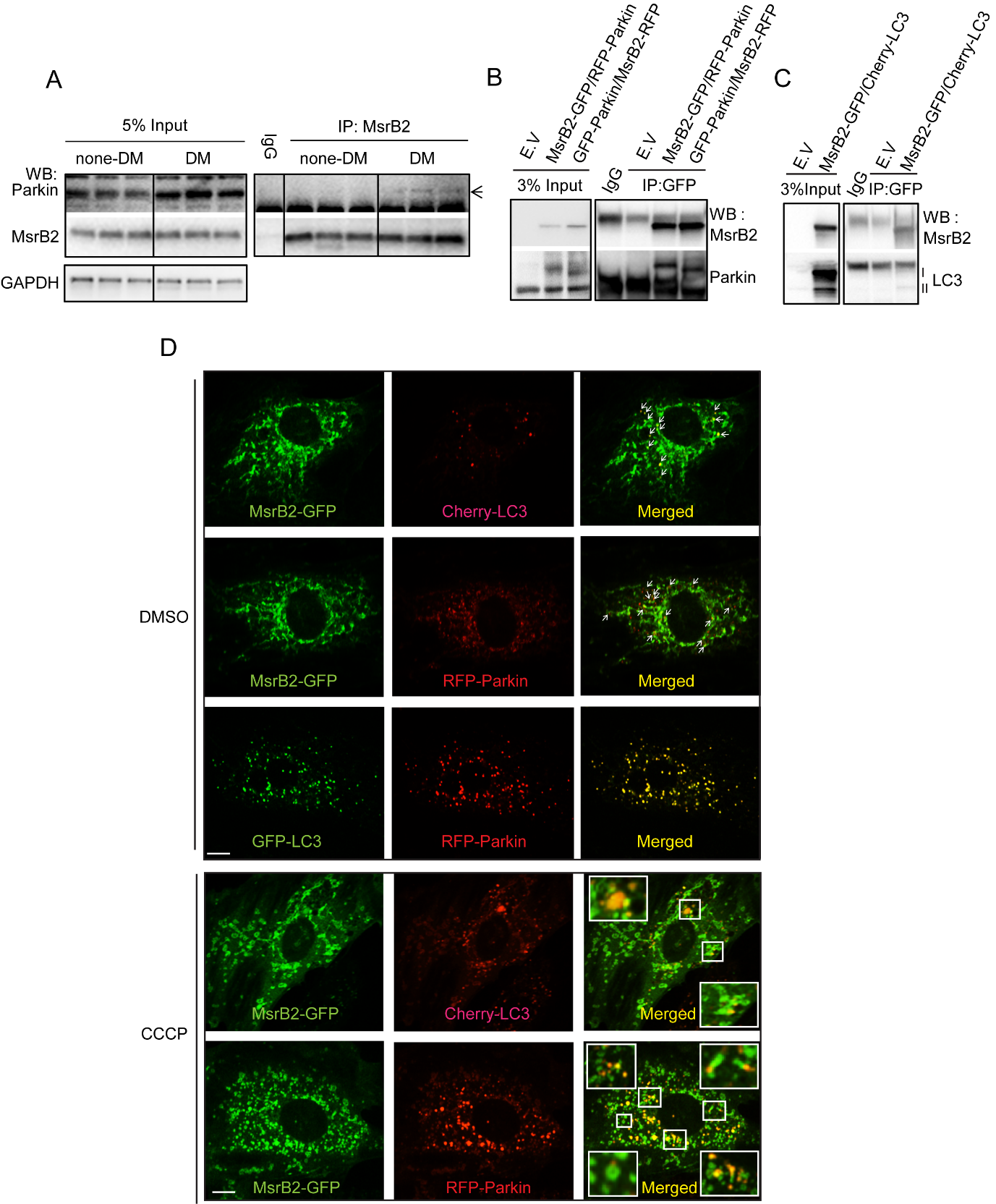
**

**Additional File 8**

**
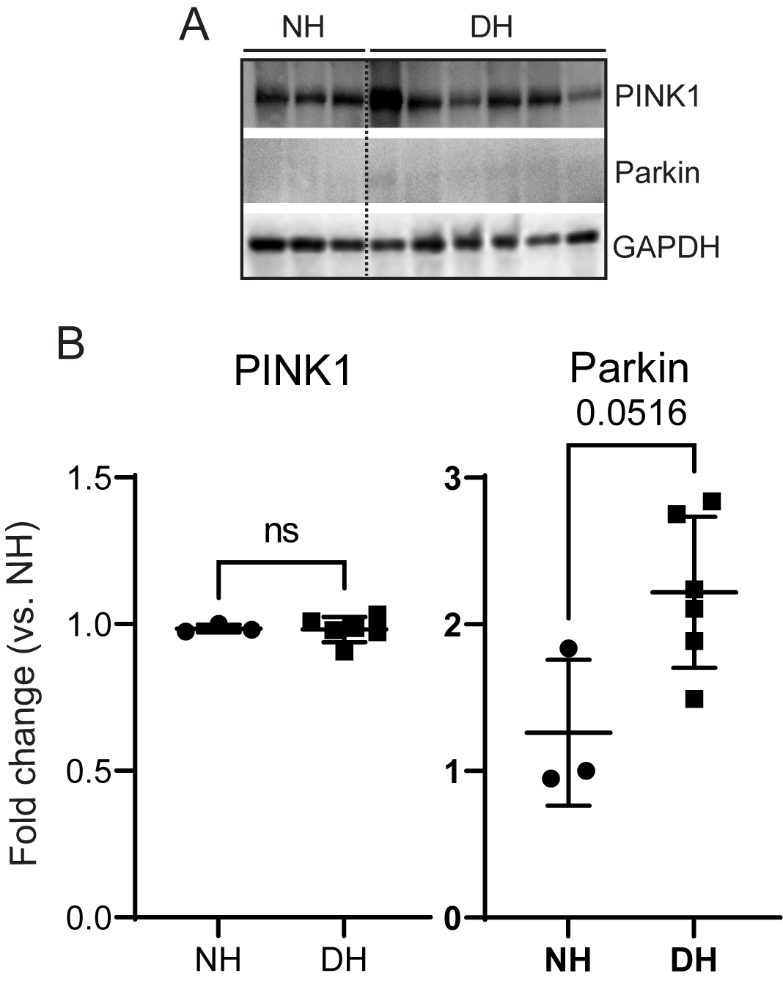
**
